# Affect Labeling During Pictorial Encoding Enhances Their Recognition and Reduces Amygdalar Responses to Negative Pictures

**DOI:** 10.1002/brb3.71297

**Published:** 2026-02-28

**Authors:** Huiyan Lin, Xiaokang Jin, Hua Jin

**Affiliations:** ^1^ Laboratory For Behavioral and Regional Finance Guangdong University of Finance Guangzhou China; ^2^ Institute of Applied Psychology, School of Psychology and Entrepreneurship Education Guangdong University of Finance Guangzhou China; ^3^ Key Research Base of Humanities and Social Sciences of the Ministry of Education, Academy of Psychology and Behavior Tianjin Normal University Tianjin China; ^4^ Faculty of Psychology Tianjin Normal University Tianjin China

**Keywords:** affect labeling, amygdala, memory, negative pictures

## Abstract

**Introduction:**

Affect labeling, which entails identifying the emotional content of an event, is a critical strategy for implicit emotion regulation. Previous studies have shown that affect labeling influences the encoding memory stage of negative events. However, it remains unclear whether affect labeling during encoding of negative events affects subsequent memory stages related to these events, such as retrieval.

**Methods:**

The current behavioral and functional magnetic resonance imaging (fMRI) study aimed to investigate whether affect labeling during encoding of emotional pictures influenced behavioral performance and neural responses during recognition memory of these pictures. To address this issue, 37 participants in the present study were instructed to label the emotional content of negative or neutral pictures, label the person‐related content, or simply view the pictures. Participants were then re‐exposed to the pictures along with several new ones and were asked to indicate whether they had previously seen each prompted picture.

**Results:**

Behavioral results showed that affect labeling during pictorial encoding subsequently increased recognition performance across all pictures. fMRI results revealed that during the encoding phase, negative pictures in the affect labeling condition elicited greater amygdala activation compared to the person labeling and viewing conditions, with this effect being even more pronounced relative to neutral pictures. More importantly, during the recognition phase, prior affect labeling reduced amygdalar responses specifically to negative pictures, which made comparable the responses observed for neutral pictures.

**Conclusion:**

These findings suggest that affect labeling during encoding of negative events modulates their memory formation, thereby influencing subsequent retrieval of those events.

## Graphical abstract

Our study suggests that affect labeling during encoding of negative stimuli decreases amygdalar activations during recognition of those stimuli.

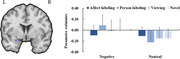

## 1 Introduction

Emotion regulation, which enables us to respond adaptively to emotional events, facilitates coping with stress and maintaining mental health. Emotion regulation can be categorized into explicit and implicit emotion regulation (Gyurak et al. 2011). Explicit emotion regulation involves conscious effort to initiate and requires ongoing monitoring during implementation (Gyurak et al. 2011). In contrast, there is a growing focus on implicit emotion regulation, which occurs without insight or awareness and is completed without monitoring (Gyurak et al. 2011). One frequently investigated strategy of implicit emotion regulation is affect labeling. This strategy involves putting self‐relevant and self‐irrelevant emotional experiences into words, which can be done through several approaches, for example, selecting from provided emotion labels (Torre and Lieberman 2018). Affect labeling could not only change individuals' emotional reactions to relevant events, but also alter how the events are encoded (Burklund et al. 2014; Constantinou et al. 2013, Constantinou et al. 2015, Constantinou et al. 2014; Cuthbert et al. 2000; Creswell et al. 2007; Critchley et al. 2000; Fan et al. 2019; Gorno‐Tempini et al. 2001; Habel et al. 2007; Hajcak et al. 2006; Hariri et al. 2000; Herbert et al. 2013; Levy‐Gigi and Shamay‐Tsoory 2022; Liang and Lin 2023; Lieberman et al. 2011, Lieberman et al. 2007; Scheuerecker et al. 2007; Tupak et al. 2014; Vives et al. 2021). Encoding, which is thought to be the first stage of memory, might influence later stages, such as retrieval. Therefore, the question of interest is how affect labeling influences later memory stages (e.g., retrieval) of negative events.

Previous functional magnetic resonance imaging (fMRI) studies have investigated whether affect labeling influences brain activity, particularly in the amygdala, during the encoding of negative events. The amygdala is involved in emotional responsiveness, attention, stimulus salience, and so forth. (Costafreda et al. 2008; Fusar‐Poli et al. 2009a, 2009b; Liu et al. 2021; Qiu et al. 2022; Sabatinelli et al. 2011; Sergerie et al. 2008). Previous studies have shown that both positive and negative stimuli activate the amygdala (Garavan et al. 2001; Hamann and Mao 2002; Lin et al. 2020, Lin et al. 2016). Furthermore, studies have indicated that amygdalar activity to negative events is altered by several explicit emotion regulation strategies (Etkin et al. 2015; Heatherton 2011; McRae 2016; Morawetz et al. 2017; Ochsner and Gross 2008). For example, cognitive reappraisal aimed at enhancing emotional responses increases amygdalar activation in response to negative pictures, while reappraisal aimed at reducing emotional responses decreases the activation (Lin et al. 2021; Lin and Liang 2023; Rotshtein et al. 2010).

Regarding the neural effects of affect labeling, research has produced mixed findings. Some studies have reported increased amygdalar activation in response to negative events under affect labeling (Gorno‐Tempini et al. 2001; Habel et al. 2007; Vives et al. 2021). Conversely, several other studies have shown that affect labeling reduces amygdalar responses to relevant events (Creswell et al. 2007; Critchley et al. 2000; Hariri et al. 2000; Lieberman et al. 2007; Torrisi et al. 2013; Vives et al. 2021). Additionally, some studies have found no significant effect of affect labeling on amygdalar responses to negative events (Lin et al. 2021; Scheuerecker et al. 2007). These discrepancies might be attributed to factors such as the duration of blood‐oxygen‐level‐dependent (BOLD) response recordings. Our recent event‐related potential (ERP) study (Lin and Liang 2023) found that affect labeling increased late positive potential (LPP) responses to negative pictures within 3–4 s, but decreased them thereafter. The LPP response is thought to reflect amygdalar activity to some extent (Rotshtein et al. 2010; Schindler and Kissler 2016; Sabatinelli et al. 2013). Therefore, shorter BOLD response recordings might be relevant to increased amygdalar responses, while longer recordings might be associated with reduced or combined responses.

Note that although the abovementioned fMRI studies have not investigated how affect labeling influences amygdalar responses to neutral pictures, our ERP study (Liang and Lin 2023) suggested that affect labeling enhanced LPP responses to neutral pictures within a relatively short duration. While this effect was smaller than that observed for negative pictures, it might imply that affect labeling elevates amygdalar responses to neutral pictures to some extent within short BOLD response recordings.

Taken together, the above‐described studies might suggest that affect labeling influences amygdalar activity during stimulus encoding, and this effect is stronger for negative stimuli compared to neutral stimuli. However, to the best of our knowledge, no studies have investigated whether affect labeling during stimulus encoding influences subsequent memory stages of these stimuli, such as retrieval. Affect labeling involves enhanced awareness and attention toward emotional content (Levy‐Gigi and Shamay‐Tsoory 2022; Torre et al. 2018). On one hand, previous studies have shown that focusing on the emotional aspects of stimuli can lead to deeper encoding of relevant stimuli. This deeper encoding has been found to improve subsequent recognition performance not only for emotional stimuli but also for neutral ones (Talmi et al. 2008). On the other hand, heightened awareness and attention can facilitate the search for strategies to reduce emotional responses and distress, especially when individuals are faced with highly intense negative stimuli (Levy‐Gigi and Shamay‐Tsoory 2022). When relevant stimuli are re‐exposed, individuals may experience reduced emotional responses and distress, which can be reflected in diminished LPP responses (Liang and Lin 2023; Lin and Liang 2023). As noted earlier, the LPP is believed to be linked to activity in the amygdala. Accordingly, affect labeling during stimulus encoding might also increase subsequent recognition performance for all stimuli and decrease amygdalar responses specifically for negative stimuli.

Accordingly, the present study aimed to investigate whether affect labeling during pictorial encoding influences behavioral and neural responses during subsequent recognition memory of these pictures. To address this issue, participants were asked to label the emotional content of negative or neutral pictures during the encoding phase. Based on previous studies (Burklund et al. 2014; Hajcak et al. 2006; Torrisi et al. 2013), participants were also required to label whether there were persons within the pictures or to freely view the pictures, which served as control conditions of the affect labeling task. The inclusion of the person labeling condition was to determine whether the observed effects were specific to affect labeling or applicable to labeling all contents. The viewing condition was included to understand the difference between the regulation (including affect and person labeling) and no‐regulation conditions and more importantly, to further determine whether the differences between the affect and person labeling conditions are associated with affect or person labeling (e.g., the differences would be associated with affect labeling if the viewing condition differs from the affect labeling condition but not the person labeling condition, but with the person labeling if the viewing condition differs from the person labeling condition but not the affect labeling condition). Additionally, all tasks were designed to be completed within a relatively short duration to mitigate the abovementioned combined effects of affect labeling on amygdalar responses during pictorial encoding. Following the labeling of emotional and person‐related contents and the viewing of the pictures, participants entered a recognition phase. During this phase, participants were presented with previously seen pictures again and several other novel ones, and were required to indicate whether the prompted picture had been viewed before. BOLD responses were recorded during both the encoding and recognition phases.

Based on the abovementioned studies and inferences, we predicted that affect labeling would increase amygdalar responses during pictorial encoding to a greater extent than person labeling and viewing, and this effect would be stronger for negative pictures compared to neutral pictures. More importantly, during the recognition phase, we predicted that, compared to person labeling and viewing, affect labeling during pictorial encoding would result in better recognition performance for all pictures. Amygdalar activation for negative pictures in the affect labeling condition would be weaker than that for negative pictures in the person labeling and viewing conditions, having decreased to a level comparable to that for neutral pictures. Neutral pictures would exhibit consistent activation levels across all tasks.

## 2 Method

### 2.1 Participants

Forty‐six undergraduate and postgraduate students were recruited as participants from Tianjin Normal University, China, via advertisements. Six participants were excluded due to excessive movements (> 3 mm of maximum displacement in the *x*, *y*, and *z* directions and/or > 3° of angular rotation along each axis). In addition, fMRI data for the recognition phase were analyzed only for trials with accurate recognition (see the “fMRI data acquisition and analysis” section for details), and the limited number of trials may have impacted the quality of data averaging. Therefore, another three participants, who exhibited low hit rates for either old or novel pictures (< 25%), were also excluded. The exclusion criteria were determined based on the number of trials commonly used in previous fMRI studies on memory (e.g., Szucs and Ioannidis 2020). This led to a final sample of 37 participants (18–27 years, M ± SD = 20.89 ± 2.33; 17 females). For behavioral data, we considered whether this sample size could detect a small to medium effect size based on our previous study (Liang and Lin 2023). Therefore, a sensitivity power analysis (statistical test: ANOVA, repeated measures, within factors; alpha: 0.05; power: 80%; non‐sphericity correction: 1; number of measurements: 4 or 6 for the interaction between task and emotion category, and 2 for relevant pairwise‐comparisons) was performed using the G*Power software (version 3.1.7; Faul et al. 2007). The findings revealed that this sample size could detect a small to medium effect size for the interaction (*f* = 0.175–0.195) and for relevant pairwise comparisons (*f* = 0.237). fMRI analysis would require other significant thresholds and parameters. This study was regarded as preliminary and served as a pilot study and basis for future larger studies. Participants were right‐handed as determined by the Edinburgh Handedness Inventory (Oldfield 1971). All participants had normal or corrected‐to‐normal vision, and no participants had a history of neurological or psychiatric disease. The study was conducted in accordance with the guidelines of ethical standards in the Declaration of Helsinki and was approved by the Ethics Committee of Tianjin Normal University (No. 2021122403). Written informed consent was obtained from all participants prior to participation.

### 2.2 Stimuli

The stimuli included 368 color pictures (184 negative and 184 neutral; 4 negative and 4 neutral for practice). Pictures were taken from the International Affective Picture System (IAPS; Lang et al. 2005) and the Emotional Picture Set (EmoPicS; Wessa et al. 2010). The number of negative and neutral pictures taken from each database was equal. Half of the pictures contained humans (e.g., human faces and bodies), and the other half contained non‐human content (e.g., animals, objects, buildings, and scenes). All pictures were adjusted to a size of 9.00 cm × 6.74 cm (horizontal × vertical) and a resolution of 28.35 pixels/cm.

Based on the normative valence and arousal ratings of the pictures, negative pictures were rated as more unpleasant and arousing than neutral pictures (valence: negative vs. neutral: M ± SD = 2.89 ± 0.63 vs. 5.04 ± 0.28; *F*(1, 358) = 1709.31, *p* < 0.001, $\eta_{p}^{2}$ = 0.827; arousal: 6.12 ± 0.82 vs. 4.26 ± 1.03; *F*(1, 358) = 357.15, *p* < 0.001, $\eta_{p}^{2}$ = 0.499). The pictures that were presented in the actual experiment were pseudorandomly separated into four sets, with 40 negative and 40 neutral pictures for each of three sets and 60 negative and 60 neutral pictures for the remaining set. For each set, an equal number of negative and neutral pictures were taken from the IAPS, and the same applied to the EmoPicS. For each emotional category of pictures, the valence and arousal ratings of the sets were comparable (*ps* ≥ 0.884). For each emotion category in each set, half of the pictures contained humans, and the other half contained non‐human content. The three sets were used as target pictures and were presented in both the encoding and recognition phases. These sets were distributed randomly to the affect labeling, person labeling, or viewing condition. The assignments of these sets to conditions were counterbalanced across participants. The remaining set of pictures was used as novel pictures and were presented only in the recognition phase.

### 2.3 Procedure

Presentation of stimuli and recording of behavioral responses were controlled by E‐Prime 2.0 software (Psychology Software Tools, Inc., Sharpsburg, PA, USA). All stimuli were shown via a back‐projection screen onto an overhead mirror. All pictures were presented with a grey background.

**FIGURE 1:**
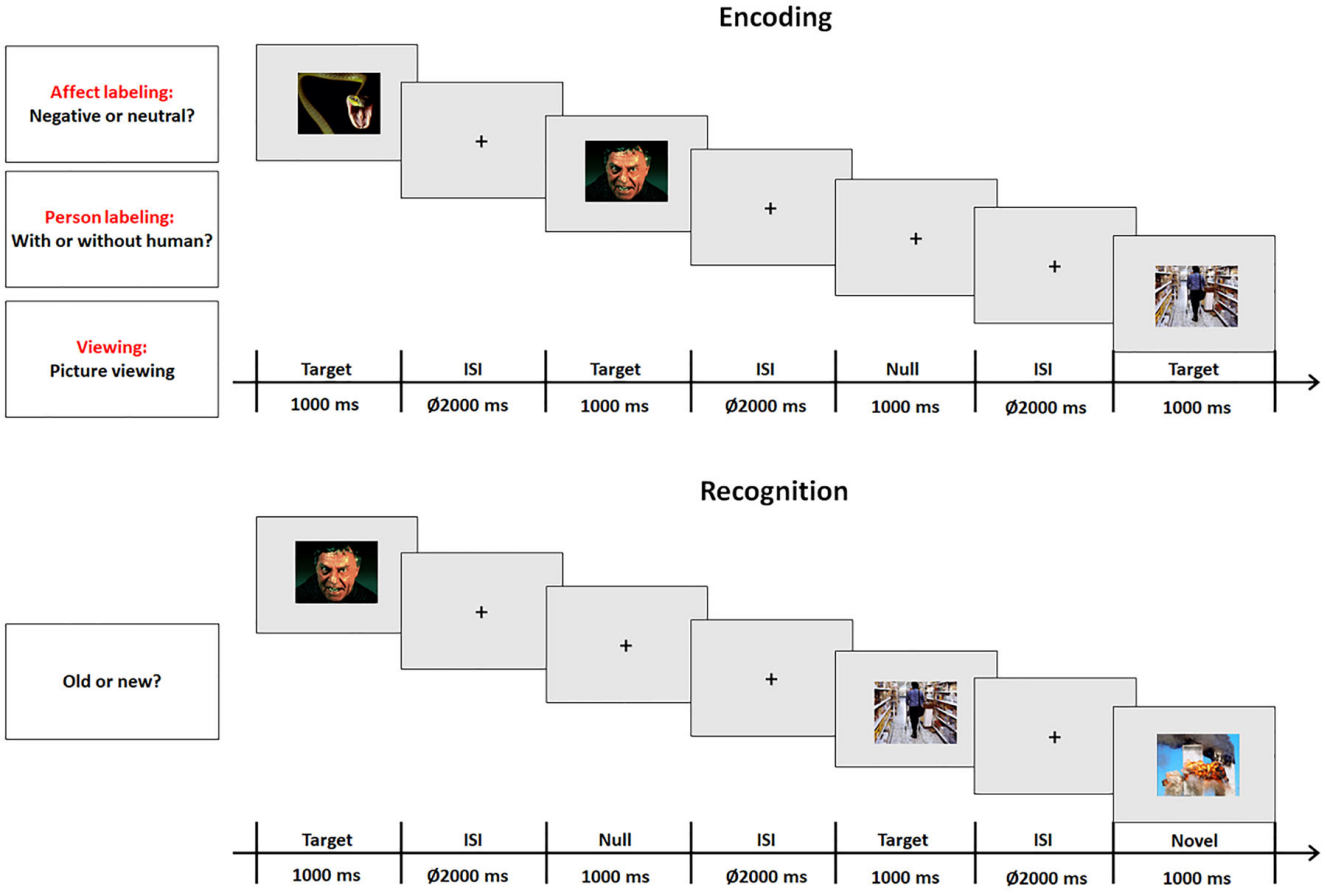
Experimental procedure in the encoding session (the upper panel) and in the recognition session (the lower panel).

As shown in Figure 1, the experiment contained two sessions, that is, the encoding and the recognition sessions. In the encoding session, MRI scanning was conducted in three runs: one in the affect labeling condition, one in the person labeling condition, and one in the viewing condition. The presentation sequence of the runs was counterbalanced across participants. In each run, each stimulus was presented once, resulting in 80 trials (40 pictures/emotion category × 2 emotion categories). Each picture was presented for 1000 ms. The presentation sequence regarding the emotion category was randomized. Interstimulus interval (ISI, the temporal interval between the offset of one stimulus and the onset of another) was 2000 ms. In the affect labeling condition, participants were required to indicate whether the presented picture displayed a negative or a neutral emotion before the presentation of the following picture via button presses of one of two buttons with the index fingers of either the left or right hand using a fiber optic response box (LUMItouch; Photon Control). For the person labeling condition, participants were required to indicate whether the picture contained humans. These two tasks emphasized accuracy and reaction times. The assignments of response buttons were counterbalanced across participants. In the viewing condition, participants were asked to view the pictures carefully and give a response by randomly pressing one of the buttons. Additionally, 16 null stimuli (a fixation cross was presented for 1000 ms and was distinguished from the fixation cross seen between the presentation of the pictures) were randomly intermixed into the sequence of pictorial stimuli per run. No tasks were required for the null stimuli. These null stimuli resulted in temporal jittering of inter‐stimulus intervals (Josephs and Henson 1999). Prior to each run, instructions were given to the participants, and the participants were then required to perform eight practice trials to familiarize themselves with the experimental procedure.

The recognition session started immediately after the encoding session. The participants did not know about this session until this point in the experiment. In this session, MRI scanning was conducted in 4 runs, with 90 trials (10 target pictures/emotion category and task × 2 emotion categories × 3 tasks + 15 novel pictures/emotion category × 2 emotion categories) for each run. Each picture was presented for 1000 ms. The presentation sequences regarding emotion category, task condition, and novelty were randomized. ISI was 2000 ms. Participants were required to indicate whether the prompted picture had been presented in the preceding encoding phase or not before the presentation of the subsequent picture. Instructions emphasized accuracy and reaction times. The assignments of response buttons were counterbalanced across participants. Additionally, 18 null stimuli were randomly intermixed into the pictorial stimuli per run. In general, the complete experiment (including the practice) lasted approximately 40 min.

### 2.4 Behavioral Data Acquisition and Analysis

Regarding the encoding session, accuracy (i.e., proportion of correct responses) and reaction times in the affect and person labeling conditions were recorded. Reaction times were analyzed for correct trials only. Accuracy and reaction times were separately analyzed with repeated measures analyses of variance (ANOVA) with within‐subject factors task (affect labeling vs. person labeling) and emotion category (negative vs. neutral).

For the recognition session, hit rates (rates of correctly identified learned pictures) and false alarm rates (rates of wrongly identified novel pictures), as well as reaction times for correct responses, were recorded. Hit rates and false alarm rates were also used to calculate the sensitivity index *d’* scores (*d’ *= *Z*_hit rates_—*Z*_false alarm rates_) and response bias (*C* = −0.5 × [*Z*_hit rates_ + *Z*_false alarm rates_]). Hit rates, *d’* scores, *C* scores, and reaction times were separately analyzed with within‐subject repeated measures ANOVA with the factor task (affect labeling vs. person labeling vs. viewing) and emotion category (negative vs. neutral). False alarm rates were analyzed with a within‐subject ANOVA with the factor emotion category (negative vs. neutral).

**FIGURE 2:**
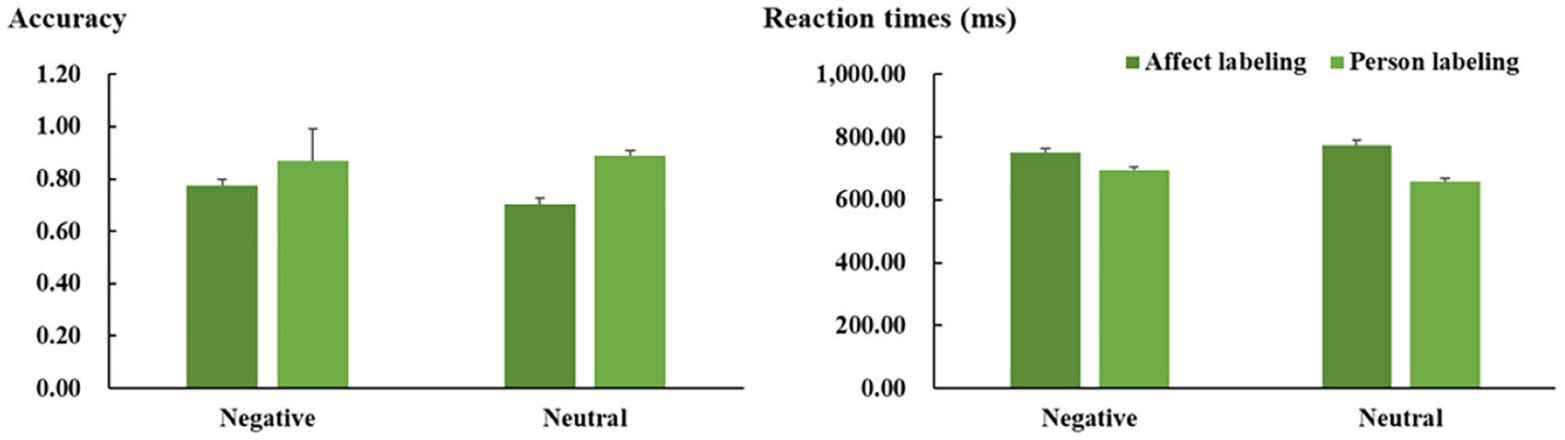
Mean accuracy (i.e., proportion of correct responses; the left panel) and reaction times (the right panel) for each experimental condition in the encoding session. Vertical lines indicate the standard error (SE) of the mean.

**FIGURE 3:**
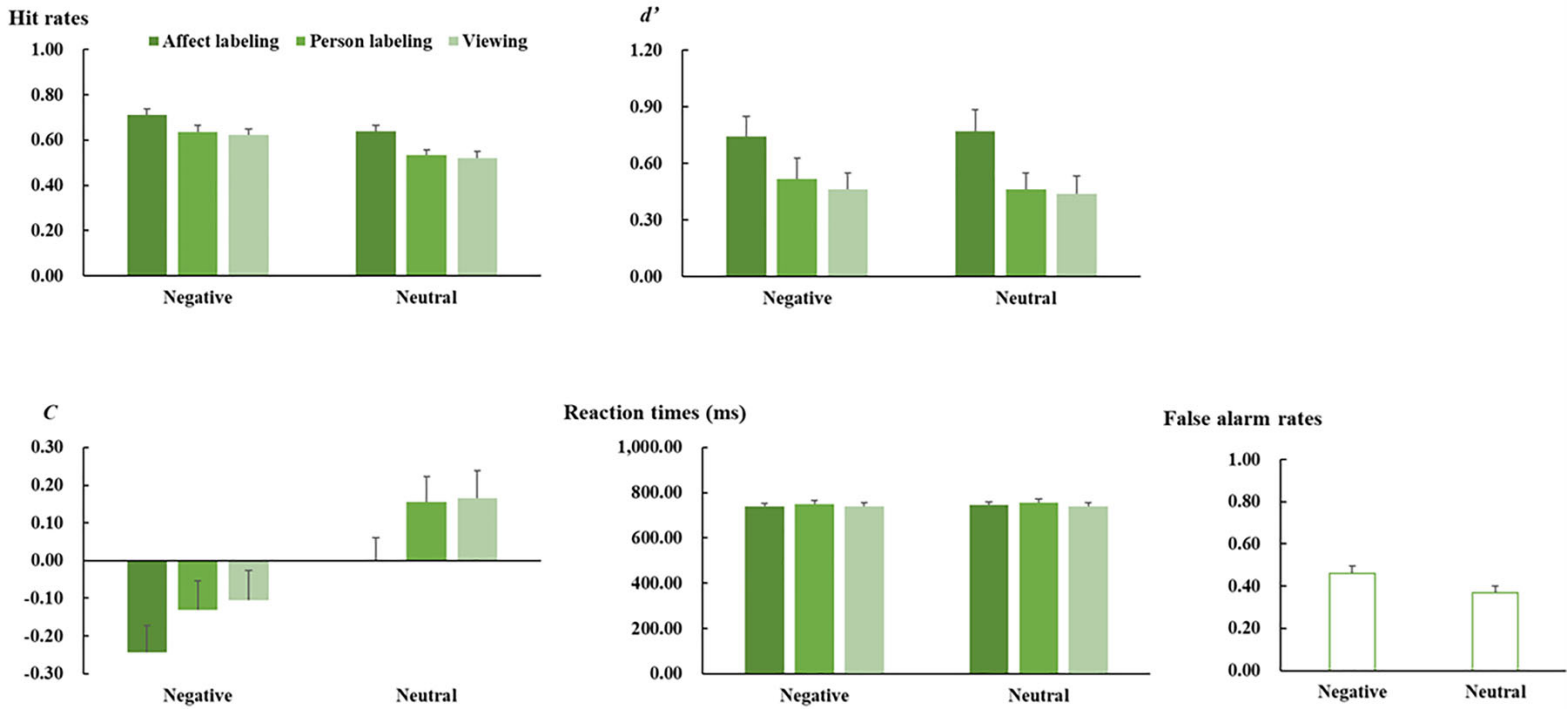
Mean hit rates (the upper‐left panel), *d’* scores (the upper‐right panel), *C* scores (the lower‐left panel), reaction times (the lower‐right panel), and false alarm rates (the lower‐ middle panel) for each experimental condition in the recognition session. Vertical lines indicate the SE of the mean.

The means and SD of behavioral data in the encoding and recognition phases are shown in Figures 2 and 3, respectively. Statistical analyses were performed using JASP 0.16.2 (https://jasp‐stats.org/). Greenhouse–Geisser corrections were applied to correct degrees of freedom and *p* values, and Bonferroni correction was used to correct post hoc *t* tests when appropriate.

### 2.5 fMRI Data Acquisition and Analysis

Structural and functional data were obtained using a 3‐Tesla magnetic resonance scanner (“MAGNETOM Prisma,” Siemens Medical Systems, Erlangen, Germany) with a 64‐channel head coil gradient set. Participants lay supine on the scanner bed and viewed stimuli back‐projected onto a screen through a mirror attached to the head coil. Foam pads were used to minimize head motion. During the tasks, blood oxygenation level‐dependent contrast functional images were acquired using a T2*‐weighted echo‐planar pulse sequence (TR = 2000 ms, TE = 30 ms, flip angle = 90°, field of view = 224 mm, matrix size = 64 × 64). For each participant, 3 runs of 146 volumes and 4 runs of 163 volumes each during the encoding and recognition tasks, respectively, were conducted. Each volume comprised 33 interleaved axial slices (thickness = 3.5 mm, gap = 0.88 mm, in‐plane resolution = 3.5 mm × 3.5 mm) orientated at an approximately 30° tilted angle from the anterior–posterior commissure plane to reduce susceptibility artifacts in the inferior parts of the anterior brain areas (Deichmann et al. 2003). The first 5 volumes of each functional run were discarded from analysis to ensure that steady‐state tissue magnetization was reached. In addition, an MP‐RAGE T1‐weighted image for each participant was also obtained (192 slices, TR = 2530 ms, TE = 2.98 ms, flip angle = 7°, field of view = 256 mm, thickness = 1 mm, gap = 0).

Functional MRI data pre‐processing and analyses were conducted using the BrainVoyager QX software package (Brain Innovation, Maastricht, the Netherlands). All volumes were realigned to the first volume to minimize artifacts due to head movements, and a slice time correction was conducted. Further data pre‐processing comprised spatial (8 mm full‐width half‐maximum isotropic Gaussian kernel) and temporal smoothing (high‐pass filter: 10 cycles/run, low‐pass filter: 2.8 s). The anatomical and functional images were co‐registered and transformed to normalized Talairach space (Talairach et al. 1988).

The preprocessed data were analyzed using multiple linear regression of the %‐standardized signal time course at each voxel. On the first level, we calculated a general linear model (GLM) with six movement parameters as nuisance regressors during the encoding phase and eight parameters during the recognition phase. We used box‐car regressors according to the onset of the pictures. A 2‐gamma hemodynamic response function was used to convolve the box‐car regressors. The movement parameters were modeled as predictors of no interest. Predictors of interest were pictures in six experimental conditions in the encoding phase (affect labeling‐negative, affect labeling‐neutral, person labeling‐negative, person labeling‐neutral, viewing‐negative, and viewing‐neutral) as well as eight conditions in the recognition phase (affect labeling‐negative, affect labeling‐neutral, person labeling‐negative, person labeling‐neutral, viewing‐negative, viewing‐neutral, novel‐negative, and novel‐neutral). Note that processing of neutral stimuli differs between true and false recognition trials (e.g., Okado and Stark 2003), a pattern that also applies to emotional stimuli (Canli et al. 1999; Marchewka et al. 2009). Previous studies on emotional memory have typically analyzed only correct‐response trials (e.g., Erk et al. 2003; Smith et al. 2004). Accordingly, the present study examined only correct‐response trials in the recognition phase to align with prior research. The mean number of valid trials per condition was as follows: affect labeling‐negative (28.50), affect labeling‐neutral (25.56), person labeling‐negative (25.38), person labeling‐neutral (21.32), viewing‐negative (24.89), viewing‐neutral (20.79), novel‐negative (32.29), and novel‐neutral (37.85). Predictor estimates were generated for each participant using a random‐effects model with adjustment for autocorrelation following a global AR(2) model. For the second level, beta values per condition were averaged for each participant with a priori‐defined regions of interest (ROIs). Analyses were conducted for the bilateral amygdala ROI as defined a priori using the Human‐Harvard–Oxford atlas (https://scalablebrainatlas.incf.org/human/HOA06). Additionally, an exploratory whole‐brain analysis was performed without a priori‐defined ROIs with the help of Neuroelf (v0.9c; http://neuroelf.net/).

Based on previous studies (Buff et al. 2017; Figel et al. 2019; Lin et al. 2024; Lin et al. 2021; Lin et al. 2020), we used a cluster‐based permutation (CBP) to correct for multiple comparisons. Significant clusters were obtained through CBP with 1000 permutations. The non‐parametric CBP framework makes no assumptions regarding the distribution of the test statistic, thereby yielding accurate false discovery rates (FDR; Eklund et al., 2016). For both the encoding and recognition phases, we modeled the main effects of emotion category (balanced contrast values: for encoding and recognition (affect labeling‐negative, affect labeling‐neutral, person labeling‐negative, person labeling‐neutral, viewing‐negative, viewing‐neutral); for recognition, additional contrasts included novel‐negative and novel‐neutral. Encoding: 1, −1, 1, −1, 1, −1 or −1, 1, −1, 1, −1, 1; recognition: 1, −1, 1, −1, 1, −1, 0, 0 or −1, 1, −1, 1, −1, 1, 0, 0) specified by weight contrasts. For the main effect of task, we modeled a difference between the affect labeling condition and the combined person labeling and viewing conditions (encoding: 2, 2, −1, −1, −1, −1 or −2, −2, 1, 1, 1, 1; recognition: 2, 2, −1, −1, −1, −1, 0, 0 or −2, −2, 1, 1, 1, 1, 0, 0). Moreover, as mentioned in the introduction section, the interaction effects might be different between the encoding and recognition phases. Therefore, we modeled different contrasts for these two phases. In the encoding phase, we modeled the interaction contrasts, which revealed higher or lower activation in the affect labeling condition versus the combined person labeling and viewing conditions for negative pictures compared to neutral pictures (i.e., 8, 4, −3, −3, −3, −3 or −8, −4, 3, 3, 3, 3). For the recognition phase, the interaction contrasts modeled stronger or weaker activation to negative pictures in the affect labeling condition compared to the combined person labeling and viewing conditions, while activations for neutral pictures were comparable across all tasks and similar to those for negative pictures in the affect labeling condition (−1, −1, 2, −1, 2, −1, 0, 0 or 1, 1, −2, 1, −2, 1, 0, 0). Voxel‐level threshold was set to *p*_uncorrected_ < 0.005. For each permutation, individual beta maps representing the activation pattern in a specific effect were randomly assigned without replacement to one of the two groups. Cluster mass was assessed by summing all *t*‐values in neighboring significant voxels. The observed cluster mass was then compared with the distribution of the maximum cluster mass observed in each of the 1000 permutations. Cluster masses larger than the 95% of the permutation distribution were considered statistically significant.

Additionally, we further explored relationships between amygdalar responses and behavioral data by computing correlations between beta values for encoding‐ and recognition‐related amygdalar activity and behavioral indices in the encoding (accuracy, reaction times) and recognition phases (hit rates, *d′* scores, *C* scores, reaction times). For each emotion category, we computed encoding‐related behavioral differences between affect labeling and person labeling, along with differences in amygdala beta values for encoding‐ and recognition‐related activity, and then correlated these differences. We also calculated differences in recognition‐related behavioral parameters and encoding‐ and recognition‐related neural beta values between affect labeling and person labeling/viewing (or combined) conditions, and then correlated these differences. See detailed results in Tables S1–S4.

## 3 Results

### 3.1 Behavioral Data

#### 3.1.1 Behavioral Data in the Encoding Phase

Regarding accuracy, ANOVA revealed a significant main effect of task (*F*(1, 36) = 42.04, *p* < 0.001, $\eta_{p}^{2}$ = 0.539). Crucially, a significant interaction between task and emotion category was also observed (*F*(1, 36) = 4.27, *p* = 0.046, $\eta_{p}^{2}$ = 0.106; Figure 2). Post hoc *t* tests revealed that accuracy was generally lower for pictures in the affect labeling condition compared to those in the person labeling condition. Furthermore, the difference was smaller for negative pictures (*t* = 3.11, *p* = 0.016, *Cohen's d* = 0.70) than for neutral pictures (*t* = 6.04, *p* < 0.001, *Cohen's d* = 1.36).

Similarly, ANOVA on reaction times also revealed a main effect of task (*F*(1, 36) = 60.37, *p* < 0.001, $\eta_{p}^{2}$ = 0.626), which was further qualified by a 2‐way interaction between task and emotion category (*F*(1, 36) = 28.28, *p* < 0.001, $\eta_{p}^{2}$ = 0.440; Figure 2). Post hoc *t* tests revealed that reaction times were generally longer for the pictures in the affect labeling condition compared to those in the person labeling condition, with the difference being less pronounced for negative pictures (*t* = 4.50, *p* < 0.001, *Cohen's d* = 0.72) than for neutral pictures (*t* = 9.34, *p* < 0.001, *Cohen's d* = 1.49).

#### 3.1.2 Behavioral Data in the Recognition Phase

In terms of hit rates, the analysis revealed main effects of task (*F*(2, 72) = 20.77, *p* < 0.001, $\eta_{p}^{2}$ = 0.366, Figure 3) and emotion category (*F*(1, 36) = 29.73, *p* < 0.001, $\eta_{p}^{2}$ = 0.452). Post hoc *t*‐tests revealed that hit rates were generally higher for pictures in the affect labeling condition compared to those in the person labeling (*t* = 5.92, *p* < 0.001, *Cohen's d* = 0.55) and viewing conditions (*t* = 5.44, *p* < 0.001, *Cohen's d* = 0.63), with no significant difference between the person labeling and viewing conditions (*p* = 1.000). In addition, hit rates were higher for negative pictures compared to neutral pictures. The interaction between task and emotion category was not significant (*p* = 0.415).

ANOVA on *d’* scores revealed a significant main effect of task (*F*(2, 72) = 19.20, *p* < 0.001, $\eta_{p}^{2}$ = .348, Figure 3). The scores were generally larger for the pictures in the affect labeling condition than for those in the person labeling (*t* = 4.99, *p* < 0.001, *Cohen's d* = 0.43) and viewing conditions (*t* = 5.68, *p* < 0.001, *Cohen's d* = 0.49), with not significant difference between the person labeling and viewing conditions (*p* = 1.000). The other main effect and interaction were not significant (*p* ≥ 0.532).

With respect to *C* scores, the analysis revealed main effects of task (*F*(2, 72) = 19.20, *p* < 0.001, $\eta_{p}^{2}$ = 0.348, Figure 3) and emotion category (*F*(1, 36) = 31.38, *p* < 0.001, $\eta_{p}^{2}$ = 0.466). In general, the scores were smaller for the pictures in the affect labeling condition compared to those in the person labeling and viewing conditions (*t* = 5.72, *p* < 0.001, *Cohen's d* = 0.31 and *t* = 5.38, *p* < 0.001, *Cohen's d* = 0.35, respectively), and the difference between the person labeling and viewing conditions did not reach statistical significance (*p* = 1.000). The scores were also smaller for negative pictures compared to neutral pictures. The difference between task and emotion category was not significant (*p* = 0.532).

ANOVA on false alarm rates revealed a main effect of task (*F*(1, 36) = 22.35, *p* < 0.001, $\eta_{p}^{2}$ = 0.383, Figure 3), with higher rates for negative pictures compared to neutral pictures. In addition, the analysis of reaction times did not show any significant main effects or interaction (*p* ≥ 0.060).

### 3.2 fMRI Results

#### 3.2.1 ROI Analysis

**FIGURE 4:**
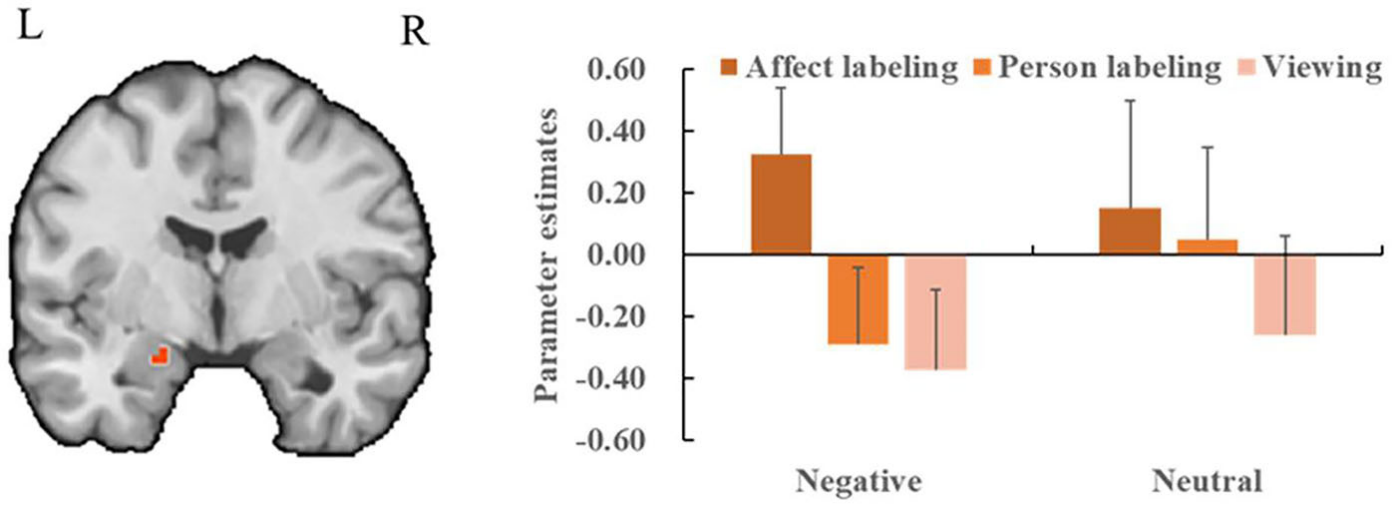
Left: The region of interest (ROI) analysis of neural activity in bilateral amygdala examines the interaction between task and emotion category during the encoding phase. The orange region indicates a significant cluster in the left amygdala, with greater activity in the affect labeling condition relative to the combined person labeling and viewing conditions for negative pictures compared to neutral pictures (*p* < 0.05, CBP corrected). Right: Bar plots display mean beta values and their SEs for the significant cluster in the left amygdala across each experimental condition.

In the encoding phase, the task contrast revealed a significant cluster in the left amygdala, with higher activations for pictures in the affect labeling condition than for the pictures in the combined person labeling and viewing conditions (*x* = −19, *y* = −4, *z* = −19; *t*_max_ = 3.35, *p* < 0.05, CBP corrected; cluster size = 81 voxels). Crucially, the interaction contrast revealed a significant cluster in the left amygdala, showing stronger activation in the affect labeling condition compared to the combined person labeling and viewing conditions for negative pictures compared to neutral pictures (*x* = −19, *y* = −4, *z* = −19; *t*_max_ = 3.36, *p* < 0.05, CBP corrected; cluster size = 81 voxels; Figure 4). Other contrasts showed no significant results.

**FIGURE 5:**
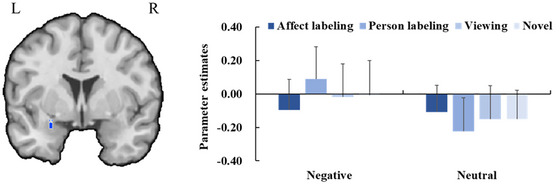
Left: The region of interest (ROI) analysis of neural activity in bilateral amygdala examines the interaction between task and emotion category during the recognition phase. The orange region indicates a significant cluster in the left amygdala, with decreased activity for negative pictures in the affect labeling condition compared to those in the combined person labeling and viewing conditions and for neutral pictures irrespective of task (*p* < 0.05, CBP corrected). Right: Bar plots display mean beta values and their SEs for the significant cluster in the left amygdala across each experimental condition.

In the recognition phase, activation in the left amygdala was weaker when comparing pictures in the affect labeling condition to the combined person labeling and viewing conditions (*x* = −24, *y* = −1, *z* = −28; *t*_max_ = 3.62, *p* < 0.05, CBP corrected; cluster size = 84 voxels). More importantly, the activation of the left amygdala for negative pictures in the affect labeling condition was lower than that for negative pictures in the combined person labeling and viewing conditions, having dropped to a level comparable to that for neutral pictures across task conditions (*x* = −27, *y* = 2, *z* = −19; *t*_max_ = 3.31, *p* < 0.05, CBP corrected; cluster size = 43 voxels; Figure 5). No other contrasts showed significant results.

#### 3.2.2 Exploratory Whole Brain Analysis

As shown in Table S5, the encoding phase revealed clusters, which showed weaker activations for pictures in the affect labeling condition compared to those in the other conditions, in parts of the frontal, parietal, and temporal cortex, cingulate regions, and caudate, whereas a significant cluster in the fusiform gyrus showed stronger activations. Clusters in parts of the frontal, parietal, and occipital cortex and posterior cingulate reflected increased activations for negative compared to neutral pictures, while clusters in parts of the frontal gyrus and cuneus were associated with decreased activations.

Additionally, a significant cluster in parahippocampal gyrus responded greater activations for negative compared to neutral pictures in the influence of affect labeling versus person labeling and viewing, whereas there were also several brain regions, including parts of frontal, parietal and temporal gyri, the cingulate and the striatum (i.e., caudate and putamen), which responded weaker activations for negative compared to neutral pictures in the influence of affect labeling versus the combined conditions of person labeling and viewing.

As illustrated in Table S6, the recognition phase revealed a significant cluster in the cingulate gyrus, which reflected greater activations for the pictures in the affect labeling condition compared to those in the other conditions, whereas clusters in the fusiform gyrus and superior parietal lobule were associated with weaker activations. Significant clusters, which responded with greater activations for negative compared to neutral pictures, were revealed in parts of the frontal, parietal, and temporal cortex, limbic areas, and thalamus, whereas a significant cluster in the caudate revealed a weaker response for negative pictures. Additionally, clusters in the middle occipital gyrus responded to weaker activations for negative pictures in the affect labeling versus person labeling, with activation levels for negative pictures in the affect labeling condition being weakly comparable to those for neutral pictures in both labeling conditions.

## 4 Discussion

The present study investigated the influence of affect labeling during pictorial encoding on recognition memory for these pictures. The findings revealed that during the encoding phase, affect labeling led to heightened amygdalar responses to pictures to a greater extent than person labeling and viewing, and this effect was stronger for negative pictures compared to neutral pictures. More importantly, during the subsequent recognition phase, affect labeling during pictorial encoding enhanced recognition performance for all the pictures. Affect labeling also reduced amygdalar activity for negative pictures, making it comparable to the activity observed for neutral pictures. Therefore, the findings suggest that affect labeling during stimulus encoding may influence subsequent recognition memory for negative stimuli and the associated neural activity.

### 4.1 Effects of Affect Labeling on Neural Activity During Encoding of Negative Pictures

During the encoding phase, increased amygdalar responses suggest that affect labeling enhances emotional reactivity and attention to a greater extent during the encoding of negative pictures relative to neutral pictures. Affect labeling activates pre‐existing conceptual knowledge of emotion categories (Barrett 2006). The activation involves appraising the emotional valence and intensity of the stimuli and recalling past relevant emotional experiences, which might lead to heightened emotional reactivity (Barrett et al. 2007; Pessoa and Adolphs 2010; Pessoa et al. 2005). Given that negative events are generally perceived as more unpleasant and arousing than neutral events, and that they can trigger more negative experiences compared to neutral events, emotional reactivity is heightened to a greater extent.

Moreover, affect labeling allows individuals to enhance awareness and attention toward emotional content (Levy‐Gigi and Shamay‐Tsoory 2022). Accordingly, in the present study, heightened amygdalar activation may also be attributable to increased attention allocated to negative pictures in the affect labeling condition. Nevertheless, as mentioned in the introduction section, the increased amygdalar responses might be attributed to the fact that BOLD responses were recorded immediately after affect labeling. Longer recording durations might result in decreased amygdalar activations or might not activate the amygdala at all.

Additionally, exploratory whole‐brain analysis provides evidence for stronger activations in memory‐relevant brain regions, such as the parahippocampal gyrus in the affect labeling condition compared to the other conditions, and this difference is stronger for negative relative to neutral pictures. The literature has suggested that the amygdala has extensive connections with relevant brain regions, such as projections to hippocampus and parahippocampal areas (Phelps 2004). Previous studies have shown that the hippocampus and parahippocampal are associated with memory formation, and the activity of the hippocampus and parahippocampal gyrus during stimulus encoding positively predicts subsequent retrieval performance (Alkire et al. 1998; Cameron et al. 2001). In the present study, increased activations in the parahippocampal gyrus might be relevant to heightened amygdalar activations, given the anatomical connections between these brain regions. Increased activations in the parahippocampal gyrus might reflect memory formation, potentially leading to enhanced subsequent recognition memory.

Exploratory whole‐brain analysis also revealed that several other memory‐relevant brain regions (e.g., middle temporal gyrus; Bakker‐Marshall et al. 2018; Laufer et al. 2011) and emotion regulation‐related regions (e.g., medial frontal gyrus; Etkin et al. 2011; Waugh et al. 2014) showed reduced activations in the affect labeling condition compared to the other conditions, with this effect being stronger for negative relative to neutral pictures. Taken together, the findings suggest that amygdalar responses likely reflect broader network‐level interactions with these brain regions during stimulus encoding, rather than isolated amygdala‐specific effect. Moreover, the concurrent involvement of regions supporting emotion processing (e.g., amygdala), emotion regulation (e.g., medial frontal gyrus), and memory (e.g., parahippocampal and middle temporal gyrus) indicates that affect labeling may influence emotional memory formation through distributed neural networks integrating regulatory and mnemonic processes.

Furthermore, affect labeling is a critical strategy for implicit emotion regulation. Accordingly, the whole‐brain findings might also suggest that emotion regulation mechanisms influence memory formation. Moreover, such memory formation‐related mechanisms may shape subsequent memory processes, such as recognition.

### 4.2 Effects of Affect Labeling During Encoding on Recognition Performance and Corresponding Neural Activity to Negative Pictures

During the recognition phase, the findings revealed that the affect labeling condition yielded superior response‐bias‐free recognition performance (i.e., higher *d’* scores) compared to other conditions. Affect labeling increases attention to emotional content (Levy‐Gigi and Shamay‐Tsoory 2022). It has been reported that focusing on emotional content of the stimuli in the encoding phase subsequently increases recognition performance of relevant stimuli, irrespective of stimulus emotional content (Talmi et al. 2008). In the context of the current study, affect labeling might have directed participants’ attention toward the emotional content within the pictures. This direct attention might enhance the encoding of the pictures, consequently improving later recognition performance.

Furthermore, as mentioned earlier, affect labeling might improve memory formation, although the effect was more pronounced for negative pictures compared to neutral pictures. This enhanced memory formation might also lead to better recognition performance when participants were later re‐exposed to relevant stimuli (Alkire et al. 1998; Cameron et al. 2001).

Nevertheless, we cannot rule out the influence of response bias for negative stimuli (see Limitations section for details) on the effect of affect labeling—in particular, the emotion‐dependent effect of affect labeling—on recognition performance. This response bias for negative stimuli likely stems from high task difficulty, similarity between old and new stimuli, and long experimental durations (inducing fatigue) of the present study. Future studies should examine the effects of affect labeling by reducing task difficulty, increasing old/new stimulus discriminability, and shortening sessions. Additionally, the recognition task was performed immediately after the encoding phase. It is unknown whether the effect of affect labeling during encoding on recognition memory for emotional stimuli occurs in a delayed recognition task, which should be further investigated in future studies.

With respect to neural activity, the current findings revealed that affect labeling during the encoding of negative pictures reduced amygdalar activity during their recognition, bringing it to levels comparable to neutral pictures across task conditions. These findings might suggest that affect labeling during the encoding of negative stimuli decreases emotional reactivity and attention during their recognition. The findings might be in line with previous studies, which have shown reduced physiological (e.g., skin conductance and ERP) responses to negative events when affect labeling is completed (Kircanski et al. 2012; Liang and Lin 2023; Mendolia and Kleck 1993; Niles et al. 2015). However, as the recognition task followed immediately after encoding, whether affect labeling reduces neural responses long after its completion requires further investigation.

A model of affective adaptation (Wilson and Gilbert 2008) suggests that when individuals encounter emotional events, focusing on emotional content facilitates search strategies for regulating relevant emotional content, leading to weaker emotional reactions. For the current study, affect labeling, which requires attention to emotional content, might also help individuals in regulating negative pictures. Consequently, when these negative stimuli were presented again during the recognition phase, the previously regulated stimuli not elicit further strong emotional responses. Consistent with this explanation, previous studies have also suggested that prior appraisal reduces attention and emotional reactions to negative stimuli upon their subsequent presentation (Paul et al. 2013; Schönfelder et al. 2014; Thiruchselvam et al. 2011).

Additionally, affect labeling is thought to involve linguistic processing, which could inhibit the processing of negative stimuli (Lieberman et al. 2007). Successful inhibition may reduce emotional reactions to negative stimuli. The inhibitory effect could persist even when the negative stimuli are re‐exposed again long after the initial affect labeling (Liang and Lin 2023). In the present study, affect labeling during stimulus encoding might have enhanced inhibitory processes during the subsequent recognition phase, leading to reduced amygdalar activity in response to negative stimuli. Supporting this explanation, exploratory whole‐brain analyses revealed that affect labeling during stimulus encoding generally increased activations in the cingulate gyrus—a brain region associated with emotional inhibition (Ochsner and Gross 2005; Roelofs et al. 2023; Seamans and Floresco 2022; Shackman et al. 2011)—during pictorial recognition. Nevertheless, whole‐brain analyses also revealed reduced activations in this brain region under the influence of affect labeling during pictorial encoding. This reduction might occur because inhibitory processes are reversed before and after affect labeling is completed, for example, inhibitory processes are reduced before affect labeling is completed but strengthened afterward (Liang and Lin 2023). BOLD responses during the encoding phase in the current study were recorded primarily during periods when affect labeling was not yet completed.

Another possibility is that affect labeling enhances attentional resources for detailed processing of negative stimuli during encoding. When the negative stimuli are presented again in the subsequent recognition phase, prior detailed processing may reduce stimulus salience and attention, thereby decreasing amygdalar activation.

### 4.3 Theoretical Contributions

In general, existing literature has suggested that affect labeling can influence the encoding of negative stimuli. Specifically, it has been observed that affect labeling initially strengthens amygdalar activations in response to negative stimuli, but subsequently leads to a decrease in activation levels (Creswell et al. 2007; Critchley et al. 2000; Gorno‐Tempini et al., 2001; Habel et al. 2007; Hariri et al. 2000; Lieberman et al. 2007; Lin et al. 2021; Scheuerecker et al. 2007; Torrisi et al. 2013; Vives et al. 2021). Consistent with these findings, our study also discovered that affect labeling, which was required to be executed within a short duration, resulted in heightened amygdalar responses, and moreover, this effect was especially stronger for negative compared to neutral pictures.

More importantly, our current study might advance the existing body of work by demonstrating that affect labeling during stimulus encoding affects not only the encoding of negative stimuli but also subsequent memory stages, such as recognition. Importantly, our findings indicate that affect labeling during stimulus encoding enhances recognition memory for all pictures and reduces amygdalar responses to correctly recognized negative pictures but not neutral pictures. Integrating the findings of previous studies with our current findings suggests that affect labeling exerts an influence on both the encoding and retrieval phases of memory for negative events.

Beyond amygdalar responses, we also observed altered activations in temporal regions (e.g., parahippocampal gyrus and middle temporal gyrus) and fronto‐cingulate regions (e.g., medial frontal gyrus and cingulate gyrus). The findings indicate that the observed amygdalar modulations likely reflect distributed network‐level dynamics involving memory‐related and regulatory systems, rather than isolated amygdala‐specific mechanisms.

Moreover, previous studies have demonstrated that explicit emotion regulation strategies, such as reappraisal and distraction, applied during initial exposure of negative stimuli, modulate responses during subsequent exposure even after regulation ceases. These effects particularly include altered retrieval memory of negative stimuli (Ahn et al. 2015; Denny et al. 2015; Flores and Berenbaum 2017; Katsumi and Dolcos 2020; Yeh et al. 2020). These effects arise because explicit strategies influence memory encoding and memory formation of relevant negative stimuli, yielding effects on subsequent memory processes (e.g., retrieval). However, little is known about whether implicit emotion regulation strategies during the encoding of negative stimuli similarly influence the retrieval memory of relevant stimuli when those strategies are no longer engaged. Our present study demonstrated that implicit emotion regulation strategies during encoding—particularly affect labeling—alter neural activity in brain regions linked to memory encoding and formation (e.g., amygdala, parahippocampal gyrus, and middle temporal gyrus) during initial exposure of negative stimuli. These changes in encoding‐related neural activity subsequently influence behavioral (e.g., recognition performance) and neural responses (e.g., cingulate gyrus and amygdala) during later memory stages. Taken together, previous and current studies demonstrate that, similar to explicit emotion regulation strategies, implicit ones during stimulus encoding influence subsequent memory processing of negative stimuli even after regulation ceases.

### 4.4 Limitations and Future Directions

Finally, it is important to acknowledge some limitations of the present study and make suggestions for future studies. First, the findings did not reveal emotional effects on *d’* scores in the recognition phase or amygdalar responses in either the encoding or recognition phase. According to the results on false alarm rates and *C* scores, the insignificant emotional effects on *d’* scores were explained by response bias (Visser et al. 2021), in which participants judged both old and novel negative pictures as “old.” As described above, the response bias was ascribed to high task difficulty, similarity between old and new stimuli, and long experimental durations (inducing fatigue). Future studies should reduce task difficulty, increase discriminability across stimuli, and shorten experimental duration to eliminate response bias. The emotional effect on amygdalar activation is known to depend on factors such as stimulus salience and experimental designs. Based on behavioral data, several neutral pictures in the affect labeling condition were perceived as negative during the encoding phase, potentially increasing their salience and diminishing emotional effects. In addition, successful emotion regulation through affect labeling and possibly even person labeling might have further reduced these emotional effects. Second, as noted earlier, the present study investigated the effects of affect labeling on emotional stimulus memory using an immediate recognition task where the stimuli were recognized immediately after the encoding phase. Accordingly, the current findings primarily reflect short‐term memory dynamics. Moreover, these immediate memory processes are distinct from delayed memory processes, which may involve additional consolidation mechanisms. Distinguishing immediate from delayed memory processes would help us further understand how affect labeling influences memory of emotional stimuli. Therefore, future studies could employ both immediate and delayed recognition tasks (e.g., recognizing stimuli immediately and 1 week after the encoding phase) to further investigate the related issue. Third, based on previous studies (Erk et al. 2003; Smith et al. 2004), the recognition phase in the present study analyzed only trials with correct responses. Nevertheless, a reduced number of valid trials compromises statistical power, and this approach may also lead to variability in the number of retained trials across participants. Future research could boost hit rates—for instance, by reducing task difficulty, enhancing stimulus discriminability, and shortening experimental duration—to improve statistical power and minimize inter‐participant differences in retained trial counts for further investigation of this issue. Finally, it should also be noted that the effects of affect labeling in the present study were obtained under specific experimental manipulations (e.g., affect labeling implemented via putting self‐irrelevant emotional experiences into words) and control conditions (e.g., person labeling and passive viewing). Future studies are therefore needed to examine whether similar effects extend to other affect labeling manipulations (e.g., labeling self‐relevant experiences) and alternative control conditions (e.g., affect matching).

## 5 Conclusion

The current study found that during the encoding phase, affect labeling was associated with increased amygdalar responses to negative pictures, with an even stronger effect observed for neutral pictures. During the recognition phase, affect labeling during pictorial encoding was accompanied by enhanced recognition performance for all pictures. Affect labeling was also associated with decreased amygdalar activity for negative pictures, leading to comparably low activity observed for neutral pictures. Taken together, the findings suggest that affect labeling during encoding of negative events may modulate memory encoding and formation processes of relevant events, thereby influencing subsequent retrieval‐related processes. Importantly, these effects were observed under specific experimental manipulations of affect labeling and particular control conditions employed in the present study, and future research is needed to determine whether they may generalize across different labeling manipulations and control conditions.

## Author Contributions

**Huiyan Lin**: conceptualization, methodology, investigation, supervision, funding acquisition, writing – original draft, writing – review and editing. **Xiaokang Jin**: formal analysis, investigation, writing – review and editing. **Hua Jin**: conceptualization, methodology, writing – review and editing, formal analysis, supervision.

## Funding

This work was supported by the Characteristic Innovation Projects in Universities in Guangdong Province (No. 2023KTSCX092), the 14th Five‐Year Plan Research Project for the Development of Philosophy and Social Sciences in Guangzhou (No. 2025GZQN33), the Planned Project of Philosophy and Social Science of Guangdong Province (No. GD23XXL05), the Research Program for Humanities and Social Science granted by the Ministry of Education in China (No. 21YJC190006, 23YJC190026), and the Key Research Platform Project for Universities in Guangdong Province (No. 2022WZJD005, 2024ZDZX4054).

## Ethics Statement

The study was approved by the Ethics Committee of Tianjin Normal University (No. 2021122403).

## Consent

All participants provided written informed consent in line with the standard ethical guidelines of the Declaration of Helsinki.

## Conflicts of Interest

The authors declare no competing interests.

## Supporting information




**Supporting Information**: brb371297‐sup‐0001‐SuppMat.docx

## Data Availability

The data that support the findings of this study are available from the corresponding author upon reasonable request.
