# Supplementary material for: Affect Labeling During Pictorial Encoding Enhances Their Recognition and Reduces Amygdalar Responses to Negative Pictures
Source: Brain Behav. 2026 Feb 28;16(3):e71297. doi: 10.1002/brb3.71297 (PMC12949723; doi:10.1002/brb3.71297)
Supplement: Supplementary file 1 — Supporting Information: brb371297‐sup‐0001‐SuppMat.docx [file BRB3-16-e71297-s001.docx]

**Supplemental materials**

**Supplementary Table 1.** Correlations between encoding-relevant behavioural performance and encoding/recognition-relevant amygdalar responses for the affect labeling and person labeling conditions in emotion category.

| Emotion category | Task | Beta values for amygdalar responses | Accuracy | | Reaction times | |
| --- | --- | --- | --- | --- | --- | --- |
|  |  |  | *r* | *p* | *r* | *p* |
| Negative | Affect labeling | Encoding | .192 | .254 | -.211 | .210 |
|  |  | Recognition | -.071 | .677 | -.046 | .785 |
|  | Person labeling | Encoding | -.023 | .892 | -.103 | .544 |
|  |  | Recognition | -.153 | .367 | -.241 | .150 |
| Neutral | Affect labeling | Encoding | -.062 | .714 | -.011 | .948 |
|  |  | Recognition | .063 | .711 | -.114 | .502 |
|  | Person labeling | Encoding | .249 | .138 | -.165 | .331 |
|  |  | Recognition | .026 | .877 | -.215 | .201 |

**Supplementary Table 2.** Correlations between encoding-relevant behavioural performance and encoding/recognition-relevant amygdalar responses for affect labeling vs. person labeling in emotion category.

| Emotion category | Beta values for amygdalar responses | Accuracy | | Reaction times | |
| --- | --- | --- | --- | --- | --- |
|  |  | *r* | *p* | *r* | *p* |
| Negative | Encoding | .223 | .185 | -.176 | .298 |
|  | Recognition | -.285 | .088 | -.199 | .237 |
| Neutral | Encoding | .001 | .997 | -.070 | .680 |
|  | Recognition | -.174 | .303 | .003 | .984 |

**Supplementary Table 3.** Correlations between recognition performance and encoding/recognition-relevant amygdalar responses in each experimental condition.

| Emotion category | Task | Beta values for amygdalar responses | Hit rates | | *d'* scores | | *C* scores | | Reaction times | |
| --- | --- | --- | --- | --- | --- | --- | --- | --- | --- | --- |
|  |  |  | *r* | *p* | *r* | *p* | *r* | *p* | *r* | *p* |
| Negative | Affect labeling | Encoding | .049 | .773 | -.219 | .193 | -.204 | .225 | -.085 | .617 |
|  |  | Recognition | -.051 | .763 | .254 | .129 | .229 | .174 | .123 | .467 |
|  | Person labeling | Encoding | .020 | .908 | .045 | .793 | .084 | .621 | -.096 | .573 |
|  |  | Recognition | .095 | .577 | -.070 | .682 | -.169 | .316 | **-.368** | **.025** |
|  | Viewing | Encoding | -.095 | .577 | -.153 | .366 | .011 | .946 | .172 | .308 |
|  |  | Recognition | -.019 | .912 | .155 | .361 | .092 | .589 | .124 | .464 |
| Neutral | Affect labeling | Encoding | .096 | .570 | -.071 | .677 | -.224 | .183 | -.052 | .759 |
|  |  | Recognition | .045 | .791 | .071 | .678 | .017 | .919 | .169 | .319 |
|  | Person labeling | Encoding | -.266 | .111 | .066 | .700 | .318 | .055 | .086 | .613 |
|  |  | Recognition | -.235 | .162 | .005 | .975 | .246 | .143 | .183 | .277 |
|  | Viewing | Encoding | -.054 | .753 | .163 | .334 | .154 | .363 | .067 | .694 |
|  |  | Recognition | .261 | .118 | **.348** | **.035** | -.081 | .634 | .263 | .116 |

**Supplementary Table 4.** Correlations between recognition performance and encoding/recognition-relevant amygdalar responses for affect labeling vs. person labeling or viewing or their combinations (i.e., both person labeling and viewing) in emotional category.

| Emotion Category | Task | Beta values for amygdalar responses | Hit rates | | *d'* scores | | *C* scores | | Reaction times | |
| --- | --- | --- | --- | --- | --- | --- | --- | --- | --- | --- |
|  |  |  | *r* | *p* | *r* | *p* | *r* | *p* | *r* | *p* |
| Negative | Affect vs. person labeling | Encoding | .081 | .633 | -.036 | .832 | .034 | .841 | .306 | .066 |
|  |  | Recognition | -.023 | .891 | .001 | .996 | < .001 | 1.000 | **-.463** | **.004** |
|  | Affect labeling vs. viewing | Encoding | -.142 | .400 | -.150 | .375 | .151 | .373 | -.020 | .907 |
|  |  | Recognition | .127 | .455 | .129 | .447 | -.127 | .455 | -.044 | .794 |
|  | Affect labeling vs. combinations | Encoding | -.002 | .992 | -.054 | .751 | .056 | .742 | .181 | .283 |
|  |  | Recognition | .034 | .843 | .05 | .768 | -.051 | .764 | **-.386** | **.018** |
| Neutral | Affect vs. person labeling | Encoding | .002 | .993 | .005 | .978 | -.005 | .976 | -.210 | .212 |
|  |  | Recognition | .005 | .975 | .027 | .872 | -.028 | .868 | -.080 | .637 |
|  | Affect labeling vs. viewing | Encoding | -.173 | .306 | -.139 | .412 | .141 | .404 | -.002 | .989 |
|  |  | Recognition | -.226 | .179 | -.231 | .170 | .232 | .166 | .132 | .438 |
|  | Affect labeling vs. combinations | Encoding | -.046 | .786 | -.033 | .848 | .031 | .857 | -.202 | .230 |
|  |  | Recognition | -.197 | .242 | -.192 | .254 | .192 | .256 | -.103 | .543 |

**Supplementary Table 5.** Significant activations for main effects of task and emotion category and their interaction during the encoding phase.

| Regions | Laterality | Talairach coordinates of peak voxel | | | *k* | *t*_(max)_ |
| --- | --- | --- | --- | --- | --- | --- |
|  |  | x | y | z |  |  |
| **The main effect of task** | | | | | | |
| ***Affect labeling > person labeling + viewing*** | | | | | | |
| Fusiform gyrus | R | 30 | -40 | -17 | 33 | 3.73 |
| ***Affect labeling < person labeling + viewing*** | | | | | | |
| Middle temporal gyrus | R | 54 | -22 | -8 | 68 | 3.51 |
|  | L | -54 | -4 | -17 | 69 | 3.67 |
| Precuneus | R | 36 | -67 | 31 | 24 | 3.41 |
|  |  | 9 | -46 | 28 | 459 | 4.64 |
| Middle frontal gyrus | R | 27 | 14 | 37 | 37 | 4.01 |
| Sub-gyral | R | 24 | 5 | 52 | 11 | 3.02 |
| Medial frontal gyrus | R | 18 | 32 | 34 | 13 | 3.10 |
|  | L | -6 | 53 | 10 | 13 | 2.94 |
| Anterior cingulate | R | 15 | 38 | 7 | 13 | 3.15 |
|  |  | 3 | 20 | -8 | 33 | 3.43 |
| Cingulate gyrus | R | 3 | -13 | 31 | 12 | 3.23 |
| Caudate | R | 12 | 11 | 16 | 41 | 3.79 |
| Superior frontal gyrus | L | -21 | 68 | 4 | 17 | 3.12 |
| Inferior parietal lobule | L | -45 | -70 | 52 | 20 | 3.59 |
| Angular gyrus | L | -45 | -70 | 31 | 18 | 3.21 |
| **The main effect of emotion category** | | | | | | |
| ***Negative > neutral*** | | | | | | |
| Inferior occipital gyrus | R | 36 | -76 | -2 | 465 | 4.29 |
|  |  | 33 | -97 | -8 | 11 | 3.40 |
| Precuneus | R | 21 | -52 | 31 | 68 | 3.67 |
| Superior frontal gyrus | L | -12 | 53 | 34 | 41 | 3.64 |
| Posterior cingulate | L | -30 | -73 | 7 | 13 | 3.30 |
| Middle occipital gyrus | L | -48 | -70 | -5 | 337 | 4.44 |
| ***Negative < neutral*** | | | | | | |
| Middle frontal gyrus | R | 30 | 41 | 13 | 76 | 4.25 |
| Superior frontal gyrus | R | 21 | 50 | -11 | 13 | 2.99 |
| Cuneus | L/R | 0 | -97 | 13 | 19 | 3.12 |
| **The interaction between task and emotion category** | | | | | | |
| ***Negative (affect labeling > person labeling + viewing) > neutral (affect labeling > person labeling + viewing)*** | | | | | | |
| Parahippocampal gyrus | R | 27 | -40 | -10 | 34 | 3.86 |
| ***Negative (affect labeling < person labeling + viewing) > neutral (affect labeling < person labeling + viewing)*** | | | | | | |
| Middle temporal gyrus | R | 61 | -43 | -9 | 13 | 3.30 |
|  |  | 59 | -14 | -5 | 11 | 2.96 |
|  |  | 53 | -26 | -1 | 68 | 3.49 |
|  |  | 37 | -70 | 27 | 20 | 3.36 |
|  | L | -61 | -18 | -6 | 67 | 3.67 |
| Inferior parietal lobule | R | 47 | -66 | 41 | 13 | 3.79 |
|  | L | -43 | -68 | 45 | 22 | 3.61 |
| Middle frontal gyrus | R | 27 | 12 | 35 | 36 | 3.83 |
|  | R | 27 | 3 | 48 | 21 | 3.17 |
| Anterior cingulate | R | 18 | 37 | 7 | 14 | 3.21 |
| Caudate | R | 13 | 10 | 17 | 41 | 3.81 |
| Precuneus | R | 12 | -86 | 47 | 13 | 3.39 |
| Posterior cingulate | R | 9 | -48 | 25 | 407 | 4.62 |
| Medial frontal gyrus | L | -8 | 51 | 8 | 13 | 2.90 |
| Putamen | L | -16 | 15 | -8 | 20 | 3.13 |

*Regions listed were thresholded with p < .05 (CBP corrected).*

**Supplementary Table 6.** Significant activations for main effects of task and emotion category and their interaction during the recognition phase.

| Regions | Laterality | Talairach coordinates of peak voxel | | | *k* | *t*_(max)_ |  |
| --- | --- | --- | --- | --- | --- | --- | --- |
|  |  | x | y | z |  |  |  |
| **The main effect of task** | | | | | | | |
| ***Affect labeling > person labeling + viewing*** | | | | | | | |
| Cingulate gyrus | R | 21 | -52 | 25 | 13 | 3.76 |  |
| ***Affect labeling < person labeling + viewing*** | | | | | | |  |
| Fusiform gyrus | R | 45 | -22 | -20 | 11 | 3.21 |  |
| Superior parietal lobule | R | 33 | -67 | 55 | 11 | 3.51 |  |
| **The main effect of emotion category** | | | | | | | |
| ***Negative > neutral*** | | | | | | | |
| Inferior temporal gyrus | R | 45 | -70 | 1 | 101 | 3.57 |  |
| Inferior frontal gyrus | R | 27 | 23 | -26 | 11 | 3.58 |  |
| Fusiform gyrus | R | 24 | -55 | -8 | 280 | 4.42 |  |
|  | L | -21 | -67 | -8 | 126 | 3.63 |  |
|  |  | -39 | -61 | -5 | 168 | 4.24 |  |
|  |  | -42 | -28 | -20 | 43 | 3.89 |  |
| Lentiform nucleus | R | 12 | -7 | -2 | 74 | 3.85 |  |
| Middle temporal gyrus | L | -60 | 8 | -26 | 20 | 2.99 |  |
| Inferior parietal lobule | L | -66 | -28 | 28 | 21 | 3.86 |  |
| ***Negative < neutral*** | | | | | | |  |
| Caudate | L/R | 0 | 2 | 13 | 14 | 3.82 |  |
| **The interaction between task and emotion category** | | | | | | |  |
| ***Affect labeling-negative = affect labeling/person labeling/viewing-neutral > person labeling/viewing-negative*** | | | | | | |  |
| No significant results | | | | | | |  |
| ***Affect labeling-negative = affect labeling/person labeling/viewing-neutral < person labeling/viewing-negative*** | | | | | | |  |
| Middle occipital gyrus | R | 45 | -74 | 4 | 13 | 3.14 |  |
|  | L | -54 | -74 | 0 | 91 | 4.09 |  |

*Regions listed were thresholded with p < .05 (CBP corrected).*
